# Hyperbaric Oxygen Therapy for Idiopathic Sudden Sensorineural Hearing Loss: Factors Affecting Benefits of Earlier Start and Longer Treatment Duration

**DOI:** 10.3390/diagnostics16040556

**Published:** 2026-02-13

**Authors:** Chun-Shih Chin, Yi-Wen Chen, Tsai-Yun Lee, Ming-Feng Wu

**Affiliations:** 1 Department of Chest Medicine, Taichung Veterans General Hospital, No. 1650 Taiwan Boulevard Section 4, Taichung 407219, Taiwan; chungshih@vghtc.gov.tw (C.-S.C.); ywchen@vghtc.gov.tw (Y.-W.C.); tyunotyun@gmail.com (T.-Y.L.); 2 Division of Pulmonary and Critical Care Medicine, Hyperbaric Oxygen Therapy Center, Department of Chest Medicine, Taichung Veterans General Hospital, No. 1650 Taiwan Boulevard Section 4, Taichung 407219, Taiwan; 3 Department of Post-Baccalaureate Medicine, College of Medicine, National Chung Hsing University, No. 145 Xingda Road, South District, Taichung 402204, Taiwan; 4 Department of Medical Laboratory Science and Biotechnology, Central Taiwan University of Science and Technology, No. 666, Buzih Road, Beitun District, Taichung 406053, Taiwan

**Keywords:** idiopathic sudden sensorineural hearing loss, hyperbaric oxygen therapy, prognosis, pure-tone audiometry

## Abstract

**Background/Objectives**: The study aimed to evaluate the efficacy of hearing gain using hyperbaric oxygen (HBO) therapy on patients with idiopathic sudden sensorineural hearing loss (ISSHL), and to provide recommendations with reference to treatment session, intervention time and the impairment severity. **Methods**: In this retrospective chart-review study, we analyzed data of ISSHL patients referred to us from the department of Ear, Nose and Throat (ENT) between January 2016 and December 2024. Hearing sensitivity improvements were assessed using pure-tone audiometry (PTA). **Results**: We found that 50.7% of patients (*n* = 148) had improved after 5 HBO sessions and 47.7% (*n* = 107) had improved after 10 HBO sessions. While no difference was found between different treatment cycles, we found treatment effects varied significantly depending on disease severity. Specifically, 64.3% of patients with profound ISSHL had improved (*p* = 0.010) after 5 sessions, and 69.2% (*p* = 0.002) after 10 sessions. Such improvements in patients with profound ISSHL were 3.681-fold larger than in those with mild to moderate ISSHL. In addition, patients who had received HBO therapy within 12 days of diagnosis showed the best response, with an odds ratio (OR) of 7.768 (95% CI: 2.785–21.664) (*p* = 0.000 *); those receiving HBO between 13 and 27 days had an OR of 3.974 (95% CI: 1.243–12.702), (*p* = 0.020 *); both groups were compared with those receiving HBO after 27 days. **Conclusions**: Patients with more severe ISSHL showed greater improvement with HBO therapy. Also, patients who started HBO therapy earlier showed better response; those who started later, like after 27 days, showed poorer or even no response at all.

## 1. Introduction

Idiopathic sudden sensorineural hearing loss (ISSHL) is defined as having sensorineural hearing loss, with an abrupt onset, of ≥30 dB, at ≥3 consecutive frequencies lasting for >3 days [1,2,3]. Symptoms develop typically when first waking up in bed, with a sense of fullness or blockage felt in the ear. Other symptoms include tinnitus (ringing in the ears), dizziness, and vertigo. According to the World Health Organization (WHO), hearing loss is the third leading cause of years lived with disability worldwide [4]. It currently ranks as the 15th leading cause of the global disease burden and is projected to rise to 7th place by 2030, particularly in high-income countries. While ISSHL represents an acute and critical subset of this burden, its sudden onset significantly impacts work productivity and social engagement. In Taiwan, ISSHL is being diagnosed and treated more extensively, due to efforts of ENT doctors and the availability of hyperbaric oxygen (HBO) facilities. Increasingly more of these patients are treated with HBO therapy [5,6].

In the event of gaining little benefits after systemic steroid therapy, salvage therapy with intra-tympanic steroid (ITS) is typically applied concomitantly with or without HBO therapy. Such an approach of delayed HBO therapy might make one miss the golden time for effective treatment. Gülüstan et al. reported improvements in ISSHL with HBO as well as ITS therapy [7]. A meta-analysis study on refractory ISSHL reported similar results [8] but did not mention HBO combined with other therapies. A number of clinical trials had used HBO therapy for patients with ISSHL after their salvage therapy, and showed some hearing improvements [1,9,10]. Other studies, on the other hand, reported no improvement with delayed HBO therapy [11,12]. The HBO effect is generally considered greater in patients with more severe hearing losses, as well as those with low-frequency hearing [1,9,10,13]. A retrospective study has shown that HBO therapy is ineffective for ISSHL as an adjunct to corticosteroid treatment, but without emphasizing the need for early HBO intervention [12]. HBO therapy, in theory, improves hearing through enhanced oxygen supply to the inner ear [14]. Early HBO therapy has been reported to be more effective when combined with other treatments [2,5,15]. HBO therapy for salvage purposes was also reported not to improve hearing gain if started way too late [11,16,17,18].

The standard treatment protocol for ISSHL is a tapered course of high-dose systemic steroid treatment [1,19]. In recent years, ITS has become increasingly popular among otolaryngologists. Unlike systemic steroid therapy, ITS is an attractive option because it avoids the common side effects, like endocrine disturbances, such as diabetic dysregulation, osteoporosis, or weight gain [1,19]. While steroids have some therapeutic effects on ISSHL, they also have side effects. Currently, there is an unmet medical need for effective therapy for sudden-onset unilateral ISSHL [19]. A recent study by Yamamoto et al. in 2023 suggested that sufficiently large doses of corticosteroid given initially at the early phase have good hearing outcomes on ISSHL [20]. Treatment options are in general aimed at suppressing inflammation in the inner ear while increasing blood supply and oxygenation. Therefore, it seems reasonable to explore other therapy options for combination therapy, especially different mechanisms, in order to achieve better efficacy in the therapy of ISSHL [19]. However, for ISSHL patients, ethical considerations preclude the direct comparison of HBO therapy with the current standard treatments. In our hospital, ENT department routinely offers ISSHL patients a tapered course of oral corticosteroids (OCS) with or without ITS, while adding adjunctive HBO therapy as soon as possible for referred patients.

It is generally believed that (a) the earlier ISSHL therapy (including HBO) is started and (b) the longer the HBO therapy is used for >20 h cumulatively (or >20 sessions), the better the prognosis of the disease [21]. HBO therapy does not improve hearing gain if delayed [16] and is least effective if started >3 weeks after the ISSHL onset [17]. The two key factors affecting prognosis are (a) the time from onset to therapy and (b) the severity of hearing loss [22]. Our previous study also concluded that ISSHL patients starting HBO therapy within 12 days of diagnosis have improvements 6.484 times greater compared with those starting later [6]. Consistent with this, a retrospective study in 2020 also reported that HBO therapy is ineffective in patients with severe and profound ISSHL symptoms [23].

The Undersea Hyperbaric Medical Society (UHMS) included ISSHL in its approved indications, which was reported in [10]. In April 2016, the 10th European Committee for Hyperbaric Medicine (ECHM) consensus conference provided sufficient evidence to support the use of HBO therapy. For patients with acute ISSHL presented within 2 weeks of onset, HBO therapy combined with OCS and/or ITS treatment is recommended. For patients with severe or profound hearing loss (≥70 dB), HBO therapy as an adjunct to OCS and/or ITS is also considered reasonable, but only for those within their first month of symptom onset. For patients with ISSNHL presented 6 months after symptom onset, HBO therapy alone or in combination with other treatment is not recommended [24].

Based on existing clinical studies [11,12] and our own [6,10], we know that salvage (delayed) HBO therapy is ineffective, and only add-on HBO therapy helps patients. Regarding the start of HBO, we know that the earlier the better and to never apply delay treatment. It remains to be confirmed if more HBO therapy sessions result in greater improvement. If so, which types of patients require more sessions? Specifically, we also examine HBO effects on those with more severe hearing losses, or pre-existing conditions like diabetes, hypertension, and hyperlipidemia. Because there are no unlimited medical resources, we need to analyze the exact number of sessions required, like 10 or 5, and which types of patients require 10 sessions.

In brief, in the present study, we planned to provide a clear reference of recommendation on HBO therapy for ISSHL patients through objective data analysis. To this end, we would evaluate how HBO therapy affects hearing of ISSHL patients, based on the relationship between their degree of hearing gain improvement after HBO therapy, and the interval from the onset of ISSHL to the start of HBO therapy. Specifically, we would compare the efficacy of HBO therapy courses (10 vs. 5 sessions), and determine the influences of factors like gender, age, initial degree of hearing loss, and pre-existing conditions like diabetes, hypertension, and hyperlipidemia. We would also determine whether different degrees of hearing loss, mild to moderate (<60 dB HL), severe (61 to 80 dB HL), and profound (≥81 dB HL), can all be improved through HBO therapy. Finally, we determine how the interval time from the onset of ISSHL to HBO therapy affects prognosis.

## 2. Materials and Methods

### 2.1. Study Design, Setting and Population

We retrospectively reviewed medical records of ISSHL patients between January 2016 and December 2024. All patients were referred to our department by the ENT department, already diagnosed with ISSHL based on their condition, medical history, and relevant tests (including first pure tone audiometry, or PTA). We then provided patients with medications and HBO therapy (5 or 10 sessions). For every ISSHL patient, standard therapy (OCS and/or ITS) was already started in the ENT department, prior to referral for our adjunctive HBO therapy. For OCS, patients received, for the first 2 days, a high-dose oral prednisolone at 1 mg/kg/day, administered in the form of 30 mg twice daily. The dose was gradually tapered to a final dose of 10 mg once daily on day 7, completing a total treatment duration of 7 days. For ITS, intra-tympanic dexamethasone injections were administered every 2 days over a week-long period. Hearing condition was assessed using PTA (GSI 61 Clinical Audiometer, instrument serial number: 20010300, GS07095) at 3 time points: (a) before, (b) after 5 HBO sessions and (c) after 10 HBO sessions. We also extracted patient data regarding the following: onset time of ISSHL, age, gender, affected ear, start time of HBO therapy, number of HBO sessions received, and pre-existing conditions (like diabetes, hypertension, hyperlipidemia).

The exclusion criteria for patients were incomplete PTA data (i.e., no data before or after HBO therapy). We also excluded patients with initial hearing loss of PTA < 25 dB, not meeting the WHO classification criteria for severe hearing loss. Patients with bilateral ear injuries were further excluded due to the higher chances of comorbidities, longer medical history, and poorer response to treatment. Our study was approved by the Institutional Review Board and Ethics Committee of the hospital (Approval No.: CE25740C).

### 2.2. HBO Therapy Sessions

HBO therapy of patients was performed inside a HBO chamber (HAUX STARMED 2200, manufacturer HAUX-LIFE-SUPPORT GmbH, Karlsbad-Ittersbach, Germany), following a standard 2.5 atmosphere absolute (ATA) 95 min regimen, with two 5 min air breaks per session. To avoid middle ear barotrauma, patients were required to equalize their middle ear pressures when receiving HBO therapy, and we evaluated the effectiveness of the Valsalva and Toynbee maneuvers. Patients who were unable to equalize their middle ear pressures were referred to the otolaryngologist for tympanocentesis. A single-center 20-year study earlier reported that implementing a 5-min air break significantly reduces the chance of seizure. Therefore, evaluating and determining the appropriate patient/HBO therapy protocol helps minimize the risk of central nervous system oxygen toxicity [25].

### 2.3. Measurement of Hearing

Hearing outcomes were measured in terms of hearing gain on PTA. According to the WHO [26], hearing loss is graded according to severity as follows: Grade 0: no impairment (25 dB HL or less); Grade 1: slight impairment (26–40 dB HL); Grade 2: moderate impairment (41–60 dB HL); Grade 3: severe impairment (61–80 dB HL); and Grade 4: profound impairment (81dB or above HL). Our patients were divided into 3 larger groups according to their severity of initial hearing loss: (a) mild to moderate (≤60 dB HL); (b) severe (61–80 dB HL); and (c) profound (≥81 dB HL).

### 2.4. Statistical Analyses

To determine the effectiveness of HBO therapy on ISSHL patients, study variables were compared between those receiving 5 and 10 sessions. Improvement in PTA changes was defined as either (a) with hearing “improvement”: gain ≥ 10 dB or (b) with “no improvement”: gain < 10 dB. Factors for treatments with or without effects were also compared. Since the interpretation day is critical in assessing the effectiveness of HBO treatment on ISSHL patients [6], the time for the cut-off of ineffectiveness was set at the 3rd quarter point derived from the distribution plot of the interpretation days for all participants. Significant factors were calculated with multiple logistic regression to determine their effects. Continuous variables were expressed as mean ± standard deviation (SD), and categorical variables were represented as numbers (or percentages). An independent two-sample *t*-test was used to compare continuous variables. Categorical variables were compared with the Chi-Square test. Statistical analyses were performed using the SPSS software version 18.0 (SPSS Inc., Chicago, IL, USA) with statistical significance set at *p* < 0.05.

## 3. Results

**Figure 1:**
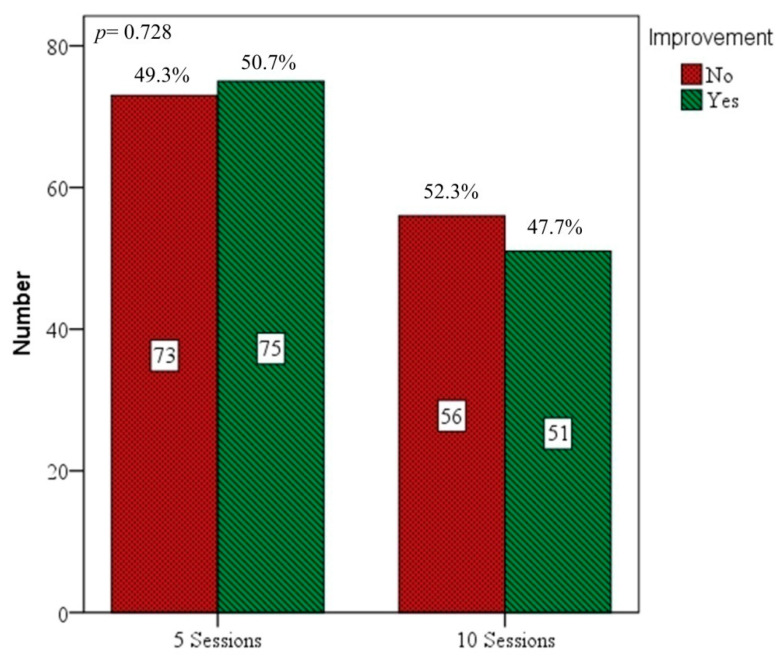
The number comparisons between patients receiving 5 and 10 sessions of HBO therapy. Hearing ‘improvement’ with hearing gain ≥ 10 dB. No improvement: <10 dB. The improvements of 5 and 10 sessions of HBO therapy were 50.7% and 47.7%, respectively (*p* = 0.728). Bars represent absolute number, with corresponding percentages (%) shown above each bar.

A total of 255 ISSHL patients (141 men, 114 women) were enrolled. Their average age was 51.3 years. Dizziness/vertigo was reported by 104 patients (40.8%), and tinnitus by 148 patients (58.0%). Of all patients, 148 received only 5 treatment sessions, and 107 received 10. Other patient characteristics are listed in Table 1, namely the start of intervention time (IT) from disease onset to the first HBO session, the total number of HBO therapy sessions received, baseline PTA data, and the severity of initial hearing loss. Hearing losses at the affected hearing frequencies were also recorded (in dB). We found no significant differences among parameters listed above between subjects receiving 5 and those receiving 10 HBO sessions. In addition, we found no significant difference in treatment improvement between the session cycles (Figure 1).

**Figure 2:**
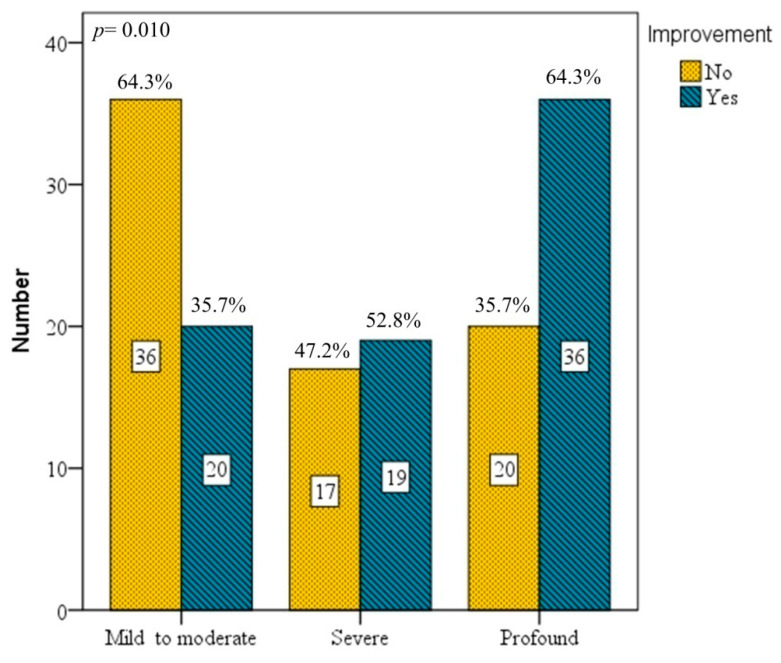
Grades of hearing impairment after 5 sessions of HBO therapy. The improvement rate for mild–moderate was 35.7%, severe 52.8%, and profound 64.3%. The treatment effect was significantly different regarding severity of initial hearing loss (*p* = 0.010). Bars represent absolute number, with corresponding percentages (%) shown above each bar.

In 148 patients receiving 5 HBO sessions, only 75 (50.7%) showed hearing improvements. Of these 148 patients, at presentation, 56 (37.8%) had mild to moderate hearing loss, 36 (24.3%) had severe hearing loss, and 56 (37.8%) had profound hearing loss. In relation to the initial severity of ISSHL, we found that the success rate of improvement was 35.7% for those with mild to moderate hearing loss, 52.8% with severe hearing loss and 64.3% with profound hearing loss. Hence, treatment efficacy varied significantly according to hearing loss severity (Figure 2). In brief, the greater the severity, the more pronounced hearing improvement was after HBO therapy.

**Figure 3:**
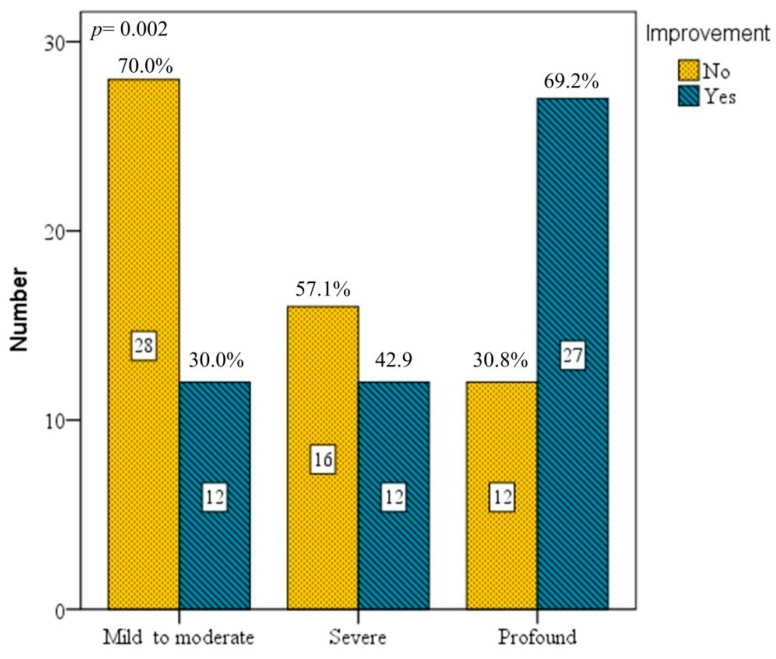
Grades of hearing impairment after 10 sessions of HBO therapy. The improvement rate for mild–moderate was 30.0%, for severe, 42.9%, and for profound, 69.2%. The treatment effect showed significant differences regarding severity of initial hearing loss (*p* = 0.002). Bars represent absolute number, with corresponding percentages (%) shown above each bar.

In 107 patients receiving 10 sessions of HBO therapy, the success rate was 30.0% for those with mild to moderate hearing loss, 42.9% with severe hearing loss, and 69.2% with profound hearing loss. Again, significant differences in treatment effectiveness existed according to disease severity (Figure 3). Results showed that 10 sessions of HBO therapy had similar improvements to those of 5 sessions. In addition, we noted that patients in both 5- and 10-session groups showed that the greater their disease severity, the more sessions they had received, and the higher their recovery rate was. This is likely related to their requirement for more HBO therapy sessions to achieve a higher success rate.

**Figure 4:**
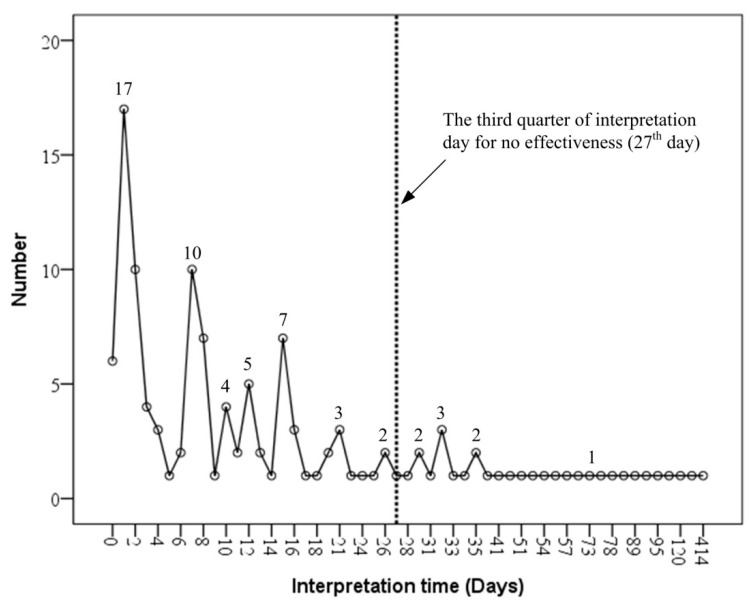
The distribution of interpretation days for patients without improvement.

**Figure 5:**
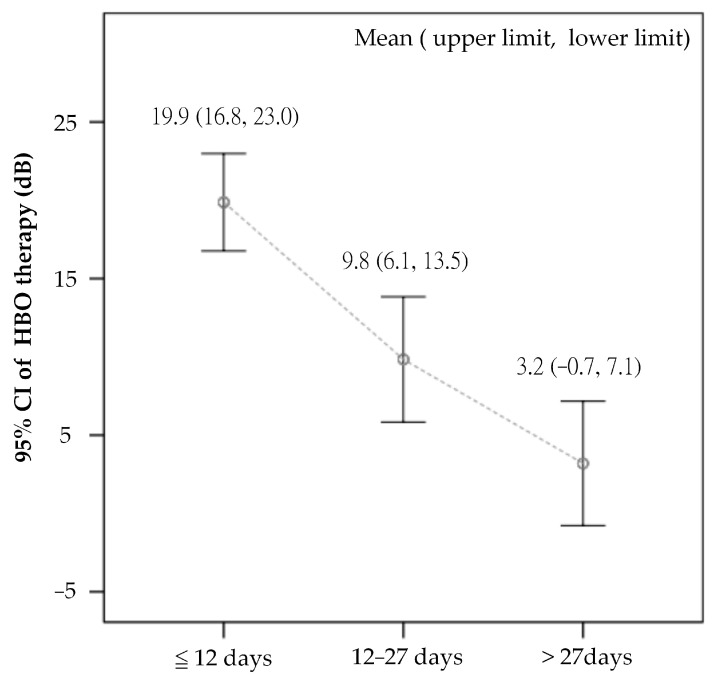
The trend of HBO therapy effects among different interpretation periods.

**Figure 6:**
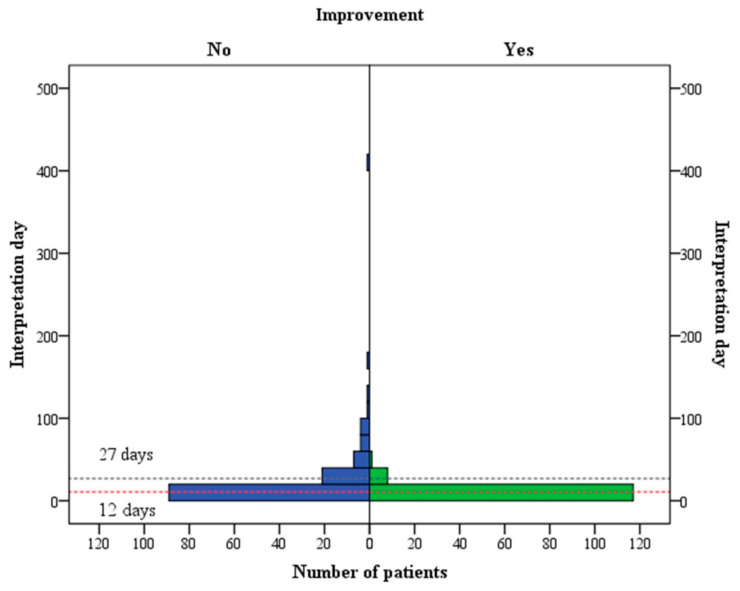
Distributions of the interpretation times (days) for patients with hearing improvement (right panel ‘yes’) or without (left panel ‘no’) after HBO treatments. The gray and red dashed lines represent the interpretation day of the 27th days and 12th days, respectively.

Furthermore, we took time intervals between the onset of ISSHL and the initiation of HBO therapy and compared them between those patients with and without hearing improvements after HBO therapy. Regarding those showing no HBO effect, they were assessed on day 27, or at the third quarter point of the distribution of interpretation days of all patients (Figure 4). In our previous study, we found that within 12 days of ISSHL onset, patients responded best to HBO therapy. Therefore, we also compared results between those who had received HBO therapy between 13 and 27 days, and those after 27 days. Therapeutic benefits of HBO therapy progressively declined as the time to initiation increased from within 12 days to beyond 27 days after disease onset (Figure 5). Initiation of HBO therapy after 27 days post-onset generated barely any benefit. Specifically, after day 27, only 4.0% of patients showed hearing improvement with HBO therapy (Table 2). The distributions of intervention time (days) of HBO therapy, for those showing some or no hearing improvement, are illustrated in Figure 6. Moreover, significant hearing gains were observed across all frequencies tested (250 Hz, 500 Hz, 1 kHz, 2 kHz, and 4 kHz) (*p* < 0.05), with the exception of 8 kHz (*p* = 0.184). For patients with hearing loss across all frequencies, the HBO effect was clearer at the lower frequencies. Note that our patients who had responded to HBO therapy (with the exception of hearing at 8 kHz) were, in general, of younger ages, started HBO therapy earlier, and had more severe initial hearing loss.

Since factors like age, interpretation day and the severity of initial hearing loss contributed significantly to the improvement of HBO therapy, we applied multiple logistic regression (MLR) analysis to assess their effects (Table 3). We compared results of patients receiving HBO therapy within 12 days against those after 27 days and patients between 13 and 27 days against those after 27 days (i.e., the intervention time from the onset of ISSHL to the start of HBO therapy). We found that the success rate of those starting HBO within 12 days had an odds ratio (OR) of 7.768 (95% CI: 1.243–12.702) compared with those starting after 27 days (*p* = 0.000 *); similarly, those starting HBO between 13 and 27 days had an OR of 3.974 (95% CI: 1.243–12.702) compared with those starting after 27 days (*p* = 0.020 *). In addition, we analyzed the hearing loss severity on effects of HBO therapy and found that patients with profound disease severity improved with an OR of 3.681 (95% CI: 1.942–6.977), much higher than those with mild to moderate disease severity (*p* = 0.000 *). On the other hand, for younger patients, multiple logistic regression revealed that HBO therapy is effective in improving hearing (OR increases by 0.979 per year of age, *p* = 0.027 *).

## 4. Discussion

According to the 10th European Hyperbaric Medicine Consensus Conference, HBO therapy is strongly recommended as the primary treatment option for ISSHL. This is supported by strong evidence, with some studies on randomized controlled trials (RCTs), with a high level of expert consensus (evidence level B). HBO therapy is typically recommended to be started within 2 weeks of ISSHL onset, and in combination with other medications, whereas it is not recommended for patients presented after 6 months of onset, HBO therapy alone or in combination with other medication. For patients presented >2 weeks but <1 month of onset, it is reasonable to apply HBO in adjunct to corticosteroid therapy, especially for those with profound hearing loss [24]. As such treatments are widely accepted and found to be beneficial in most cases, it is likely considered unethical to withhold them from HBO therapy. The three most promising treatments for ISSHL patients are (a) corticosteroids including ITS, (b) vasodilators, and (c) HBO therapy. Of these treatments, only HBO therapy has sufficient randomized controlled trials to yield positive meta-analysis results (Cochrane review) [27].

In our study, patients were referred to us after prior treatment at our ENT department. We specifically measured pure-tone hearing sensitivities before and after HBO therapy and recorded their time of intervention (from ISSHL onset to initiation of HBO therapy). Some studies have reported that HBO therapy has no benefits on hearing if delayed in application [11,17,18,21]. Other studies reported that when combined with other clinically approved treatments (systemic steroids, ITS), early HBO therapy is more effective than treatments without HBO [2,15]. In our study, all patients received tapering oral high-dose corticosteroids and/or ITS. We found that their outcomes differed depending on the different ranges of intervention time, i.e., within 12 days, between 13 and 27 days, and after 27 days. Patients who underwent early intervention within 12 days had the best outcomes than those after 27 days, with an odds ratio of 7.768. Early HBO therapy within 12 days of onset was associated with significantly larger hearing improvements. Therefore, results are in support of the choice in starting HBO therapy as early as possible. This is also in line with the recommendation at the 10th European Consensus Conference on Hyperbaric Medicine.

One study compared HBO therapy and ITS injections for hearing restoration in patients with ISSHL after failing primary treatments [7]. Similar improvements were reported between ITS and HBO in the literature. However, as the mechanisms of these two treatments are different (i.e., ITS is anti-inflammatory therapy and HBO therapy increases oxygen supply to provide the raw materials for repair), we chose to apply both treatments together to our patients. Other studies reported that HBO therapy is more effective in patients with initially severe hearing loss [1,9,10,21]. One study showed that patients with moderate ISSHL experienced improved hearing after delayed salvage HBO therapy combined with oral steroids, while HBO therapy is not effective in patients with severe or profound hearing loss [23]. Two other studies reported that HBO therapy combined with systemic steroids and ITS therapy show little hearing recovery regardless of the severity of hearing loss [12,28]. Although no difference was found, it is generally accepted that HBO therapy should be considered as a treatment strategy for this patient group, especially those with severe and profound hearing losses [28]. In summary, HBO therapy is likely more effective for patients with severe and profound hearing losses, and less so for patients with mild to moderate hearing loss. In the current study, we also considered relevant factors, including circulatory disorders such as diabetes, hypertension, and hyperlipidemia. We found that HBO therapy was more effective when initiated earlier in younger patients [6] and in those with severe or profound initial hearing loss.

A systematic review and meta-analysis concluded that the longer the duration of HBO therapy (>20 h, ~20 treatment sessions), the greater the recovery efficacy [21]. In our study, we divided the duration of HBO therapy into two groups: the 5 and 10 treatment sessions. We found that HBO therapy was equally effective in both groups, but there was no further improvement when extended beyond 10 treatment sessions. Also, we found a better improvement rate in patients with profound disease severity. Specifically, their improvement was 3.681-fold over those with mild to moderate disease. HBO therapy combined with OCS and/or ITS therapy demonstrated a synergistic effect, particularly in younger patients and those with initial hearing loss exceeding 50 dB. The therapeutic benefit was more pronounced in the low-frequency range. It is recommended to administer at least five HBO sessions [29]. There is no standard reference to define exactly when the intervention time becomes ineffective. Our previous study has shown that the later HBO is introduced, the less effective it is [6]. In that study, we identified a cutoff of 27 days as the ineffective intervention time for patients. After this time point, only 4.0% of patients responded positively to HBO. This finding provides strong evidence for an intervention timeframe. At the 10th ECHM (European Consensus Conference on Hyperbaric Medicine) consensus conference, it was recommended that HBO therapy combined with OCS and/or ITS be used for patients with ISSHL who present within 2 weeks of onset. For patients with more severe hearing loss, adjunctive HBO therapy within 4 weeks after symptom onset is considered reasonable [24]. HBO therapy is least effective if started more than 3 weeks after ISSHL onset [17]. The cochlea, an organ of the inner ear, depends on adequate oxygen levels in the blood. However, likely due to its concealed location within the temporal bone, the blood supply is very limited, akin to a terminal circulation without venous return, relying mainly on lymphatic supply. Cochlear blood is supplied through the labyrinthine artery. The high-frequency cochlear hair cells are located relatively distant from the arterial circulation, likely resulting in a less efficient response to HBO therapy. It is not surprising to find significant hearing improvements of our patients across most frequencies except the high frequency region at 8 kHz (*p* = 0.184). Since objective data are essential for providing effective advice to our patients, our study found that HBO therapy should be administered in conjunction with other treatments and initiated as early as possible, without delay.

There are some limitations of our study. First, we followed up with PTA data of two groups of patients after each completed 5 or 10 sessions of HBO therapy. However, in the real world, PTA cannot routinely be performed due to various factors, resulting in incomplete dataset. Second, we did not include bilateral sudden hearing loss in our study. Most bilateral hearing losses occur gradually over time, despite some with abrupt onset. A systematic review reported that the incidence of bilateral hearing loss is <5% [1]. The most common causes are related to aging (age-related hearing loss) or prolonged noise exposure. Other causes include (a) inner ear diseases such as Meniere’s disease and autoimmune diseases; (b) auditory nerve disorders such as acoustic neuroma, viral or bacterial infections; (c) medications like ototoxic drugs; and (d) genetic factors. To treat the above-mentioned diseases, the underlying causes must be addressed. Given unresolved causes, virtually no therapy, even HBO, is effective.

## 5. Conclusions

In our study on adjunctive HBO therapy for two groups of ISSHL patients, (a) the 5 sessions group contained 56 (37.8%) patients with mild to moderate hearing impairment before HBO therapy, 36 (24.3%) with severe hearing impairment, and 56 (37.8%) with profound hearing impairment, and (b) the 10 sessions group contained 40 (37.4%) patients with mild to moderate hearing impairment, 28 (26.2%) with severe hearing impairment, and 39 (36.4%) with profound hearing impairment. Comparing patients with profound hearing impaired and those with mild to moderate hearing impairment, we found greater improvement (3.681 fold) in those with profound hearing impairment (*p* = 0.000 *). The more severe the ISSHL, the more pronounced the effect of HBO therapy was. Hearing gains were of statistical significance across all tested frequencies (250 Hz, 500 Hz, 1 kHz, 2 kHz, and 4 kHz), with the exception of 8 kHz (*p* < 0.05). HBO therapy hence has significant beneficial improvement, particularly in the lower-frequency regions.

Comparing HBO therapy started within 12 days or started between 12 and 27 days against starting after 27 days (i.e., the time from ISSHL onset to HBO initiation), the success rate was 7.768-fold higher for those who started HBO treatment early within 12 days relative to those who started late after 27 days (*p* = 0.000 *), or 3.974-fold higher for those that started between 13 and 27 days relative to those that started after 27 days (*p* = 0.020 *). We therefore recommend that ISSHL patients should begin HBO therapy within 12 days of symptom onset and to receive at least five sessions of HBO therapy, especially for younger patients. Also, in the event of hearing loss ≥ 81 dB (profound hearing impairment), we recommend 10 sessions of HBO therapy.

## Figures and Tables

**Table 1:** Basic characteristics of enrolled patients.

	All (n = 255)	5 Sessions (n = 148)	10 Sessions (n = 107)	*p*-Value
Age (years old)	51.3 ± 14.7	51.9 ± 14.7	50.6 ± 14.9	0.484
Sex				0.173
Male	141 (55.3)	76 (51.4)	65 (60.7)	
Female	114 (44.7)	72 (48.6)	42 (39.3)	
Dizziness/Vertigo	104 (40.8)	56 (37.8)	48 (44.9)	0.319
Tinnitus	148 (58.0)	84 (56.8)	64 (59.8)	0.719
Hypertension	63 (24.7)	40 (27.0)	23 (21.5)	0.388
Hyperlipidemia	78 (30.6)	43 (29.1)	35 (32.7)	0.626
Diabetes mellitus	41 (16.1)	20 (13.5)	21 (19.6)	0.255
HBO intervention time (days)	15.6 ± 32.9	13.0 ± 36.4	19.0 ± 27.1	0.151
Baseline PTA (dB)	72.3 ± 27.8	73.1 ± 27.8	71.6 ± 27.8	0.659
Baseline severity				0.942
Mild–moderate	96 (36.6)	56 (37.8)	40 (37.4)	
Severe	64 (25.1)	36 (24.3)	28 (26.2)	
Profound	95 (37.3)	56 (37.8)	39 (36.4)	
250 Hz (dB)	61.9 ± 27.4	63.8 ± 27.6	59.3 ± 27.0	0.204
500 Hz (dB)	70.0 ± 28.6	71.2 ± 28.0	68.4 ± 29.4	0.44
1 K Hz (dB)	73.3 ± 28.8	73.4 ± 28.9	73.1 ± 28.7	0.924
2 K Hz (dB)	70.3 ± 30.5	70.5 ± 31.2	70.0 ± 29.5	0.886
4 K Hz (dB)	74.7 ± 28.9	74.6 ± 29.4	74.9 ± 28.5	0.942
8 K Hz (dB)	75.6 ± 26.5	76.0 ± 26.4	74.9 ± 26.9	0.742

PTA: pure-tone audiometry; HBO: hyperbaric oxygen; mild–moderate: 25–60 dB, severe: 61–80 dB, profound: ≥81 dB.

**Table 2:** Factor comparisons between treatment effectiveness of HBO therapy. Patients who responded to HBO therapy are those younger, those starting therapy earlier, and those with more severe hearing loss at the beginning, with the exception of 8 k Hz. Patients who started HBO therapy > 27 days after disease onset showed minimal effects.

	Improvement (N = 126)	No Improvement (N = 129)	*p*-Value
Age (years old)	49.4 ± 14.8	53.2 ± 14.5	0.036 *
Sex			0.966
Male	69(54.8)	72 (55.8)	
Female	57 (45.2)	57 (44.2)	
Dizziness/Vertigo	55 (43.7)	49 (38.0)	0.428
Tinnitus	72 (57.1)	76 (58.9)	0.873
Hypertension	31 (24.6)	32 (24.8)	1
Hyperlipidemia	40 (31.7)	38 (29.5)	0.794
Diabetes mellitus	27 (21.4)	14 (10.9)	0.033 *
HBO intervention time (days)	7.5 ± 7.8	23.4 ± 44.3	0.000 *
<12 days	104 (82.5)	72 (55.8)	
12–27 days	17 (13.5)	26 (20.2)	
>27 days	5 (4.0)	31 (24.0)	
HBO treatment sessions			0.728
5 times	75 (59.5)	73 (56.6)	
10 times	51 (40.5)	56 (43.4)	
Baseline PTA (dB)	80.1 ± 25.5	65.0 ± 27.9	0.000 *
Baseline severity			0.000 *
Mild–moderate	32 (25.4)	64 (49.6)	
Severe	31 (24.6)	33 (25.6)	
Profound	63 (50.0)	32 (24.8)	
250 Hz (dB)	65.7 ± 27.3	58.2 ± 27.0	0.030 *
500 Hz (dB)	77.3 ± 26.1	63.0 ± 29.3	0.000 *
1 K Hz (dB)	80.5 ± 26.0	66.3 ± 29.7	0.000 *
2 K Hz (dB)	77.5 ± 28.4	63.2 ± 30.8	0.000 *
4 K Hz (dB)	79.5 ± 28.4	70.0 ± 28.8	0.008 *
8 K Hz (dB)	77.8 ± 25.8	73.4 ± 27.2	0.184

PTA: pure-tone audiometry; HBO: hyperbaric oxygen; mild–moderate: 25–60 dB, severe: 61–80 dB, profound: ≥81 dB. *: *p*-value <0.05.

**Table 3:** Multiple logistic regression of PTA data on hearing improvement after HBO treatments.

Factors	Odds Ratio (95% CI)	*p*-Value
Age		
Per year of age	0.979 (0.960–0.998)	0.027 *
Diabetes mellitus		
Yes vs. no	2.071 (0.947–4.529)	0.068
Grades of hearing impairment		
Severe vs. mild–moderate	1.918 (0.960–3.831)	0.065
Profound vs. mild–moderate	3.681 (1.942–6.977)	0.000 *
HBO interpretation period		
13–27 days vs. 27 days	3.974 (1.243–12.702)	0.020 *
≤12 days vs. 27 days	7.768 (2.785–21.664)	0.000 *

PTA: pure-tone audiometry; HBO: hyperbaric oxygen; mild–moderate: 25–60 dB, severe: 61–80 dB, profound: ≥81 dB. *: *p*-value <0.05.

## Data Availability

The raw data supporting the conclusions of this article will be made available by the authors on request.

## Abbreviations

The following abbreviations are used in this manuscript:

ATA	Atmosphere absolute
ENT	Ear, Nose and Throat
HBO	Hyperbaric oxygen
IT	Intervention time
ISSHIL	Idiopathic Sudden Sensorineural Hearing Loss
ITS	Intra-tympanic steroid
OCS	Oral corticosteroids
OR	Odds ratio
UHMS	Undersea Hyperbaric Medical Society

## References

[1] Eryigit B., Ziylan F., Yaz F., Thomeer H. (2018). The Effectiveness of Hyperbaric Oxygen in Patients with Idiopathic Sudden Sensorineural Hearing Loss: A Systematic Review. Eur. Arch. Otorhinolaryngol..

[2] Khater A., El-Anwar M.W., Nofal A.A., Elbahrawy A.T. (2018). Sudden Sensorineural Hearing Loss: Comparative Study of Different Treatment Modalities. Int. Arch. Otorhinolaryngol..

[3] Tsuzuki N., Wasano K. (2024). Idiopathic Sudden Sensorineural Hearing Loss: A Review Focused on the Contribution of Vascular Pathologies. Auris Nasus Larynx.

[4] World Health Organization (2024). World Health Statistics 2024: Monitoring Health for the SDGs, Sustainable Development Goals.

[5] Wang H.H., Chen Y.T., Chou S.F., Lee L.C., Wang J.H., Lai Y.H., Chang H.T. (2023). Effect of the Timing of Hyperbaric Oxygen Therapy on the Prognosis of Patients with Idiopathic Sudden Sensorineural Hearing Loss. Biomedicines.

[6] Chin C.S., Lee T.Y., Chen Y.W., Wu M.F. (2022). Idiopathic Sudden Sensorineural Hearing Loss: Is Hyperbaric Oxygen Treatment the Sooner and Longer, the Better?. J. Pers. Med..

[7] Gülüstan F., Yazıcı Z.M., Alakhras W.M.E., Erdur O., Acipayam H., Kufeciler L., Kayhan F.T. (2016). Intratympanic Steroid Injection and Hyperbaric Oxygen Therapy for the Treatment of Refractory Sudden Hearing Loss. Braz. J. Otorhinolaryngol..

[8] Kuo T.C., Chao W.C., Yang C.H., Tsai M.S., Tsai Y.T., Lee Y.C. (2022). Intratympanic Steroid Injection Versus Hyperbaric Oxygen Therapy in Refractory Sudden Sensorineural Hearing Loss: A Meta-Analysis. Eur. Arch. Otorhinolaryngol..

[9] Ajduk J., Ries M., Trotic R., Marinac I., Vlatka K., Bedekovic V. (2017). Hyperbaric Oxygen Therapy as Salvage Therapy for Sudden Sensorineural Hearing Loss. J. Int. Adv. Otol..

[10] Chin C.S., Lee T.Y., Wu M.F. (2017). Adjunctive Hyperbaric Oxygen Treatment for Idiopathic Sudden Sensorineural Hearing Loss. Undersea Hyperb. Med..

[11] Mariani C., Carta F., Catani G., Lobina S., Marrosu V., Corrias S., Tatti M., Puxeddu R. (2023). Idiopathic Sudden Sensorineural Hearing Loss: Effectiveness of Salvage Treatment with Intratympanic Dexamethasone or Hyperbaric Oxygen Therapy in Addition to Systemic Steroids. Front. Neurol..

[12] Skarzynski P.H., Kolodziejak A., Gos E., Skarzynska M.B., Czajka N., Skarzynski H. (2023). Hyperbaric Oxygen Therapy as an Adjunct to Corticosteroid Treatment in Sudden Sensorineural Hearing Loss: A Retrospective Study. Front. Neurol..

[13] Huo Z., Cheng X., Gu J., Hong Y., Wang Z., Zhang Z. (2022). Prognostic Factors for Hearing Outcomes in Patients that Undergo Adjuvant Hyperbaric Oxygen Therapy for Sudden Sensorineural Hearing Loss. Laryngoscope Investig. Otolaryngol..

[14] Joshua T.G., Ayub A., Wijesinghe P., Nunez D.A. (2022). Hyperbaric Oxygen Therapy for Patients With Sudden Sensorineural Hearing Loss: A Systematic Review and Meta-Analysis. JAMA Otolaryngol. Head Neck Surg..

[15] Bayoumy A.B., de Ru J.A. (2019). The Use of Hyperbaric Oxygen Therapy in Acute Hearing Loss: A Narrative Review. Eur. Arch. Otorhinolaryngol..

[16] Eski E., Babakurban S., Yılmaz S., Yılmazer C., Erkan A.N., Çaylaklı F., Yılmaz İ. (2020). Comparing the Efficiencies of Hyperbaric Oxygen Therapy and Intratympanic Steroid Treatment for Sudden Hearing Loss. J. Int. Adv. Otol..

[17] KayalıDinç A.S., Çayönü M., Boynueğri S., Ünsal Tuna E., Eryılmaz A. (2020). Is Salvage Hyperbaric Oxygen Therapy Effective for Sudden Sensorineural Hearing Loss in Patients with Non-Response to Corticostreoid Treatment^1^?. Cureus.

[18] Keseroğlu K., Toptaş G., Uluat A., Bayir Ö., Çadalli Tatar E., Saylam G., Korkmaz M.H., Özdek A. (2020). Addition of Intratympanic Steroid or Hyperbaric Oxygen Treatment to Systemic Steroid Treatment in Sudden Idiopathic Sensorineural Hearing Loss Treatment, and Long-Term Results of Salvage Treatment. Turk. J. Med. Sci..

[19] Rodríguez-Izquierdo C., Herrera M., Avdiyuk A., Rodríguez-Ocaña D., Plaza G. (2025). Current Role of the Nonsteroid Treatment of Idiopathic Sudden Sensorineural Hearing Loss (ISSNHL): A Narrative Review. J. Clin. Med..

[20] Yamamoto K., Kurioka T., Ohki M., Sano H., Yamashita T. (2023). Is Repetitive Systemic Corticosteroid Therapy Effective for Idiopathic Sudden Sensorineural Hearing Loss? A Retrospective Study. Front. Neurol..

[21] Rhee T.M., Hwang D., Lee J.S., Park J., Lee J.M. (2018). Addition of Hyperbaric Oxygen Therapy vs Medical Therapy Alone for Idiopathic Sudden Sensorineural Hearing Loss: A Systematic Review and Meta-Analysis. JAMA Otolaryngol. Head Neck Surg..

[22] Toroslu T., Erdoğan H., Çağlar Ö., Güçlü O., Dereköy F.S. (2018). Comparison of Different Treatment Methods for Idiopathic Sudden Sensorineural Hearing Loss. Turk. Arch. Otorhinolaryngol..

[23] Almutairi N., Alnofal E., Algouhi A., Bamajboor A.S., Alzaher N. (2020). The Effectiveness of Hyperbaric Oxygen Therapy as Salvage Treatment for Sudden Sensorineural Hearing Loss: A Retrospective Study. Cureus.

[24] Mathieu D., Marroni A., Kot J. (2017). Tenth European Consensus Conference on Hyperbaric Medicine: Recommendations for Accepted and Non-Accepted Clinical Indications and Practice of Hyperbaric Oxygen Treatment. Diving Hyperb. Med..

[25] Costa D.A., Ganilha J.S., Barata P.C., Guerreiro F.G. (2019). Seizure Frequency in More Than 180,000 Treatment Sessions with Hyperbaric Oxygen Therapy—A Single Centre 20-Year Analysis. Diving Hyperb. Med..

[26] Humes L.E. (2019). The World Health Organization’s Hearing-Impairment Grading System: An Evaluation for Unaided Communication in Age-Related Hearing Loss. Int. J. Audiol..

[27] Murphy-Lavoie H.M., Mutluoglu M. (2022). Hyperbaric Treatment of Sensorineural Hearing Loss. StatPearls.

[28] Yücel A., Özbuğday Y. (2020). Comparison of Steroid Treatment with and without Hyperbaric Oxygen Therapy for Idiopathic Sudden Sensorineural Hearing Loss. J. Audiol. Otol..

[29] Chen Y.-C., Liu Y.-H., Kang B.-H., Yao C.-S., Chen Y.-S., Liu W.-C. (2025). Hyperbaric oxygen therapy improves the effects of systemic steroid therapy for sudden sensorineural hearing loss. Heliyon.

